# Implementing an innovative consent form: the PREDICT experience

**DOI:** 10.1186/1748-5908-3-58

**Published:** 2008-12-31

**Authors:** Carole Decker, Suzanne V Arnold, Olawale Olabiyi, Homaa Ahmad, Elizabeth Gialde, Jamie Luark, Lisa Riggs, Terry DeJaynes, Gabriel E Soto, John A Spertus

**Affiliations:** 1 Saint Luke's Mid America Heart Institute, 4401 Wornall Rd, Kansas City, MO 64111, USA; 2 University of Missouri-Kansas City, 5100 Rockhill Rd, Kansas City, MO 64110, USA; 3 Washington University in St. Louis, 660 S. Euclid Ave, St. Louis, MO 63110, USA; 4 Children's Mercy Hospital & Clinics, 2401 Gilham Rd, Kansas City, MO 64108, USA; 5 University of Chicago, 5801 S. Ellis Ave, Chicago, IL 60637, USA

## Abstract

**Background:**

In the setting of coronary angiography, generic consent forms permit highly variable communication between patients and physicians. Even with the existence of multiple risk models, clinicians have been unable to readily access them and thus provide patients with vague estimations regarding risks of the procedure.

**Methods:**

We created a web-based vehicle, PREDICT, for embedding patient-specific estimates of risk from validated multivariable models into individualized consent documents at the point-of-care. Beginning August 2006, outpatients undergoing coronary angiography at the Mid America Heart Institute received individualized consent documents generated by PREDICT. In February 2007 this approach was expanded to all patients undergoing coronary angiography within the four Kansas City hospitals of the Saint Luke's Health System. Qualitative research methods were used to identify the implementation challenges and successes with incorporating PREDICT-enhanced consent documents into routine clinical care from multiple perspectives: administration, information systems, nurses, physicians, and patients.

**Results:**

Most clinicians found usefulness in the tool (providing clarity and educational value for patients) and satisfaction with the altered processes of care, although a few cardiologists cited delayed patient flow and excessive patient questions. The responses from administration and patients were uniformly positive. The key barrier was related to informatics.

**Conclusion:**

This preliminary experience suggests that successful change in clinical processes and organizational culture can be accomplished through multidisciplinary collaboration. A randomized trial of PREDICT consent, leveraging the accumulated knowledge from this first experience, is needed to further evaluate its impact on medical decision-making, patient compliance, and clinical outcomes.

## Background

The Institute of Medicine has challenged the American healthcare system to be more patient-centered, evidence-based, and transparent–encouraging the patient to be more involved in the decision-making process [1] so as to optimally match treatment decisions with patient preferences [2]. Patients have repeatedly expressed interest in being actively involved in the decisions about their care [3-8], although the desired level of participation varies widely in routine clinical practice [9,10]. In a study of patients' preferences for involvement in decision-making and information needs when undergoing coronary angiography, we found that patients wanted to know their options and potential outcomes but also repeatedly stated they wanted information they could readily understand and apply [11]. These findings launched a series of projects designed to integrate the patient more actively in their treatment decisions at the Mid America Heart Institute.

We focused our initial efforts on the process of informed consent prior to coronary angiography. While in routine clinical practice the informed consent process has become a passive legal event, it should be comprised of an educational process leading to informed choice. To accomplish this, Brody proposed a transparency model that sees consent as a conversational process that enhances good clinical practice and patient autonomy without sacrificing appropriate legal soundness–a process that can be facilitated by a tool that includes the specific informational needs of a particular patient at a particular moment in time [12]. A reasonable disclosure of information is deemed adequate when clinician's thought processes have been rendered transparent to the patient [13].

Using this transparency model in routine clinical practice hinges upon addressing the specific decisional needs of individual patients with patient-specific data. While professional guidelines recommend a thoughtful discussion with the patient and family about the risks and benefits of each procedure [14], this can be difficult to do in the rushed atmosphere of clinical practice [6]. In addition, the amount of data and research available to clinicians is overwhelming, making it difficult for clinicians to recall all of the potential mediating factors that are applicable to specific patients' potential outcomes. Risk prediction models can aid this process and have been reported in the literature for cardiovascular diseases since the 1970s [15-21]. However, even with the existence of validated risk models, the most effective way of communicating risk and expected outcome to patients is unclear, and consequently, explicit calculations for different outcomes are rarely used in contemporary practice [22,23]. Physicians commonly provide patients an assessment of their prognosis through intuition, experience, and convenient heuristics, rather than through formal risk estimates derived from validated models [24]. Consequently, despite progress in understanding cardiovascular outcomes, accurate, valid, and meaningful prediction models are not used routinely in the management of individual patients.

To address this gap in clinical care and to improve the ability of clinicians to engage in shared decision-making that is both evidence-based and patient-centered, we sought to augment the Brody Transparency Model with patient and clinical data related to expected outcomes, and implement this model in the process of obtaining informed consent for coronary angiography. To accomplish this goal, we created the PREDICT application of the Personalized Risk Information Services Manager (ePRISM^®^) technology [25,26], which has the ability to embed patient-specific estimates of risk from validated multivariable models into individualized consent documents at the point-of-care. We applied this technology to the process of obtaining consent for coronary angiography and were able to successfully implement this change in clinical process so that PREDICT is now part of the routine process of care. This paper describes how we were able to change organizational culture in our healthcare system and the lessons we learned through the process to assist other organizations in improving their process of informed consent.

## Methods

### Creation of the information systems and user interface

**Figure 1:**
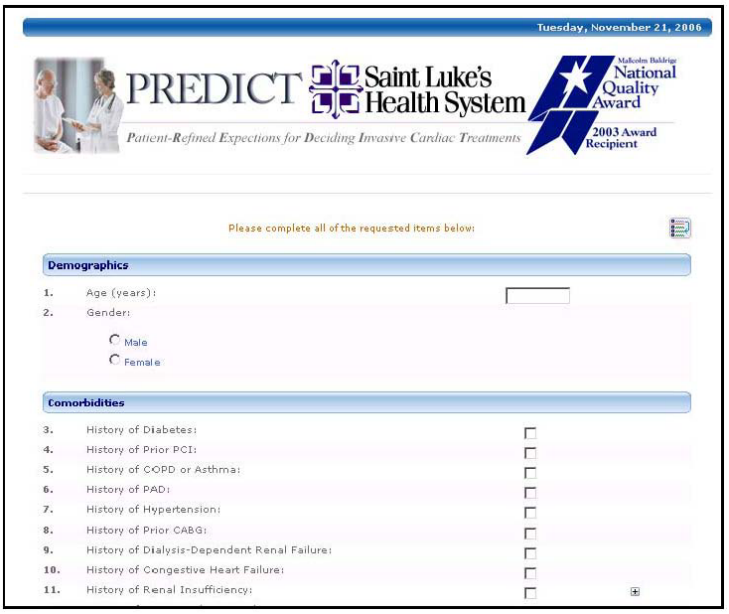
**Sample screenshot of PREDICT website**.

A multidisciplinary team developed four separate pre-procedural risk models for percutaneous coronary intervention (PCI), through access to large cardiovascular databases and collaboration with national colleagues: in-hospital mortality following PCI [19], bleeding complications following PCI [20], and one-year restenosis with bare metal and drug-eluting stents [21] (Appendix). Technology using the patient experience, complete with patient characteristics, clinical data, procedural data, and treatment options with predicted outcomes for each different selected treatment modality has been developed [25-27]. To achieve use of the enhanced consent process in clinical settings, a parsimonious set of variables needed to be established. A tool that uses a large number of variables would render the instrument too cumbersome and too time-consuming to be used in routine clinical practice, thus impeding its integration into the workflow of an office, clinic, or hospital. The final set of data to execute the four clinically relevant models, included 18 distinct patient and clinical characteristics (Table 1). To minimize data entry and the possibility of transcription errors, all patient demographic and laboratory information is automatically fed into the system in real-time from the hospital's patient registration system (see sample screenshot, Figure 1).

**Table 1:** Patient and Clinical Variables Used in PREDICT Risk Models.

**Patient characteristics**	Age
	Gender
	Body surface area
**Clinical history**	Atrial fibrillation
	Cerebral vascular accident
	Chronic lung disease
	Diabetes mellitus
	Dialysis-dependent renal failure
	Hypertension
	Peripheral artery disease
	Prior PCI
	Prior coronary artery bypass grafting
	Left ventricular ejection fraction ≤ 40%
	Serum creatinine
	Admission hemoglobin

**Disease presentation**	Primary indication for PCI*
	Priority of PCI procedure†
	Cardiac arrest at presentation

* Options include: asymptomatic coronary artery disease, stable angina, unstable angina, post-myocardial infarction angina, post-myocardial infarction anatomy, acute non-ST elevation myocardial infarction, acute ST elevation myocardial infarction, cardiogenic shock

† Options include: emergent, urgent, non-urgent

Abbreviations: PREDICT = Patient Refined Expectations for Deciding Invasive Cardiac Treatments, PCI = percutaneous coronary intervention

### Creation of the informed consent document

In addition to acquiring statistical models for outcomes, focus groups and interviews of patients recovering from myocardial infarctions were held to understand patient preferences of the best method for presentation of these risks and benefits [12]. The computer technology portion utilized both the statistical models and patient-identified visual output preferences to create ePRISM^®^. PREDICT, one application of ePRISM^® ^designed for PCI, enables the translation of available risk prediction models into routine clinical care prior to PCI [25,26]. The aims of PREDICT were two-fold: to inform patients about the procedure and their individual risks of complications so they would have more realistic expectations going into the procedure and to allow physicians to access tools that can guide clinical decisions (*i.e*., if estimated risk of bleeding is high, the physician may elect to use fluoroscopy for vascular puncture or bivalirudin as an anti-thrombotic agent). We were also hopeful that, by presenting the risks of restenosis with a bare metal and a drug-eluting stent, PREDICT would enable an informed dialogue between the patient and the interventionalist regarding type of stent to place, a decision that needs to balance the risks for restenosis with the need for prolonged dual anti-platelet therapy.

**Figure 2:**
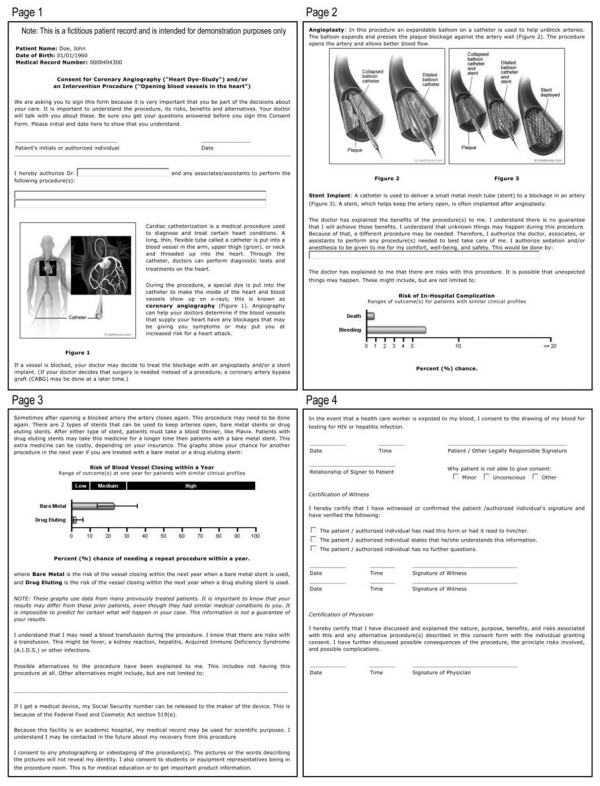
**Sample consent document generated by PREDICT with patient-specific risks of complications**.

Beyond the inclusion of the individualized statistical risk models in the consent forms, we sought to improve the consent form document in other ways to increase patients' overall understanding of the procedure. Educational pictures and descriptions of coronary catheterization, angioplasty, and stents were inserted; and the reading level of the text was reduced. We utilized the Flesch-Kincaid grade-level readability statistic [28] to ensure the consent form was written at an appropriate level of complexity for our target patients [29]. The original generic consent form was determined to be a sixteenth grade level, which is consistent with prior literacy findings of consent forms [30]. We were able to edit the new consent form to a 6.8 grade level statistic. Additionally, the use of white space, text-page placement, and font size were adjusted, as these are factors shown to be important in patient understanding [31]. Prior to use, the consent form was reviewed by risk management and legal, who required inclusion of several phrases that led to a final Flesch-Kincaid reading level statistic of 8.0 (see sample consent document, Figure 2).

### Implementation environment and strategy

Once the relevant risk models were identified, programmed, and the template of the consent form was approved, we needed to integrate PREDICT into the routine process of informing patients about the risks and benefits of PCI. PREDICT was first implemented at the Mid America Heart Institute (MAHI) in Kansas City, Missouri, with the personalized consents being generated for all outpatients undergoing coronary angiography with the possibility of PCI. Saint Luke's Hospital, which includes MAHI, is a 567-bed, university-affiliated, not-for-profit, tertiary care facility that serves as a major referral center for a 100-mile geographic area and operates five cardiac catheterization labs where approximately 1,600 PCI's were performed in 2006. Prior to initiating the new consent forms, submission to the Institutional Review Board was performed. The project was deemed to be quality improvement and a waiver of consent was granted.

After the process of creating the consent documents in this controlled environment was perfected, PREDICT was expanded to all patients undergoing non-emergent coronary angiography at the MAHI and at the three additional Kansas City metropolitan hospitals within the Saint Luke's Health System. These three additional hospitals are non-teaching institutions where the nursing staff are not as accustomed to participating in research studies. However, all of the interventional cardiologists who perform procedures at these satellite hospitals also practice at MAHI and were familiar with the PREDICT consent process at the time of the expansion. The success of implementing this new technology and enhanced consent document was assessed from the perspective of multiple participants in the healthcare system: administrators, information system staff, nurses, physicians, and patients.

### Evaluation strategy

To understand the experience with the new consent forms, a mixed methods approach was developed and used. Validated, quantitative patient-centered surveys [32] were used in a pre-post design to assess ease of reading, comprehension, and anxiety related to the consent document and process as compared to the original, generic consent document. Following the initial pilot phase (August to October 2006), qualitative data collection methods were utilized to capture clinician (both nursing and physician) perceptions through structured questionnaires, free-form interviews, and focus groups. During this time, numerous opportunities existed for staff feedback (phone, email, personal interviews, and unit supervisor reports). The experience of the information systems staff was assessed through unstructured interviews.

In addition to direct observation, data analysis was performed both individually by several team members (OO, HA, CD), and collectively through consensus meetings with all investigators and the implementation team (OO, JL, LR, EG, TD). Qualitative data analysis is typically iterative, recursive, and dynamic [33]. Moving between these two venues allowed for frequent independent reflection and then discussion by involved project members. The evolution of discrete themes could therefore be explored and either confirmed or refuted. The use of multiple reviewers enhanced the construct validity and inter-rater reliability of the coding scheme. The goal of qualitative analysis is to present a meaningful interpretation of the staff's implementation experience with PREDICT. Frequent stakeholder discussions were used to validate the themes and sub-themes that emerged from the qualitative analyses. Additionally, because a patent is pending on the ePRISM^® ^tool, safeguards to ensure objectivity in implementation, subject identification, evaluation, and interpretation were implemented. The two physicians (JAS and GES) who have a proprietary interest in ePRISM^® ^and a potential conflict of interest served as consultants to the project and were not involved in data collection or analysis.

## Results and Discussion

### Pilot testing phase

Initial pilot testing at the MAHI required numerous planning meetings with involved staff (see Implementation Timeline, Table 2). In May and June of 2006, presentations were made to senior medical and nursing leadership. These presentations, conducted by senior leadership in the project (JAS and CD), were designed to be more inspirational than logistical; with the purpose being primarily to present the overall concept and long-term goals so that necessary 'buy-in' could be secured from key opinion leaders within the organization. During this time, we identified a group of nursing 'champions,' who were in positions critical to the success of the implementation (LR: Clinical Nurse Specialist of the Cardiovascular Holding Unit; EG: Clinical Nurse Manager of the Cardiovascular Holding Unit; and JL: Cardiovascular Nurse Educator for Saint Luke's Health System), and jointly developed an implementation plan, including limiting the pilot testing to outpatients. In June and July of 2006, meetings were held with the Cardiovascular Holding Unit staff, which was the site of initial pilot testing. These meetings focused on the processes by which care would change and the necessary logistics of the project. Importantly, though, we presented the concept and rationale for the project and emphasized the importance of the staff in its successful execution.

**Table 2:** Implementation Timeline

August 2002	• Funding received from Doris Duke Foundation
	• Multidisciplinary team formed
August 2002 – December 2005	• Predictive risk models developed and validated
	• ePRISM^® ^technology created to accept input and generate patient-specific risk models
	• Patient focus groups and interviews completed (outcomes of interest, output preferences)

February 2006	• Decision made to use PREDICT in enhanced consent forms for PCI

May – June 2006	• Presentations made to senior nursing and medical leadership
	• Decision made to adopt PREDICT PCI consent form as strategic quality improvement initiative

May – June 2006	• Generic consent rewritten at lower literacy level, educational pictures and descriptions added, and risk models embedded into PCI consent form

May – July 2006	• Risk management/legal approve concept and consent form revisions

June – July 2006	• Meetings held with Cardiovascular Holding Room staff for concept introduction, input, and change initiation
	• Questionnaires created to assess patients' experiences with the informed consent process (pre- and post-PREDICT consent form)

July 2006	• Patient survey data collected pre-implementation of PREDICT consent form

August 28, 2006	• PREDICT consent form implemented for outpatients scheduled for PCI procedures
	• Patient survey data collected post-implementation of PREDICT consent form

September 2006	• Review of patient data revealed successful experience (easier to read, easier to understand, patient felt more involved in decision making, and less anxiety)
	• Focus groups/interviews of nurses, physicians, and information systems staff revealed barriers to be addressed

February 2007	• Modifications made to tool and process prior to expansion

March 2007	• PREDICT-enhanced consent for outpatient PCI procedures expanded to 3 other system hospitals in the Kansas City metropolitan area
	• System upgraded to accept real-time lab values for use in executing risk models

April 2, 2007	• PREDICT enhanced consent form expanded to include all inpatients and outpatients going for coronary angiography/PCI consent forms at Saint Luke's Health System

Abbreviations: ePRISM^® ^= electronic Personalized Risk Information Services Manager, PREDICT = Patient Refined Expectations for Deciding Invasive Cardiac Treatments, PCI = percutaneous coronary intervention

Beginning in August 2006, all outpatients with a scheduled coronary angiogram for which a PCI was possible had their informed consent customized by PREDICT (typical volume was approximately five to ten patients per day). Nursing staff in the Cardiovascular Holding Unit collected the identified patient variables that were not automatically fed into the system and entered the data over the internet into a secure server, compliant with the United States Health Insurance Portability and Accountability Act of 1996, from which the prediction models could be generated. Once the data were entered, the PREDICT program calculated the prediction models, generated a graph for each outcome (in-hospital mortality, bleeding, restenosis) and embedded these into the consent documents. The customized and enhanced PCI consent forms were printed at the nursing station and given to patients for review prior to their discussions with the physicians. Thus patients and physicians were provided with timely, convenient, and individualized patient-specific risk model information, all contained in the informed consent document that was now functioning more like a decision aid.

During the pilot testing phase, several types of feedback were available to continue to improve the process. Members of the implementation team made daily visits to the Cardiovascular Holding Unit to obtain ongoing feedback. Weekly operations meetings were held with senior medical (CD, HA) and information technology staff (TD). When issues arose with the information systems, the implementation team did troubleshooting and quickly learned to sort issues into 'internal to the hospital' issues (requiring in-hospital information technology support) versus 'PREDICT server' issues (requiring off-site information systems support [GES]). If a quick solution could not be found, the original generic consent form was used as a back-up, which occurred approximately one to two times per week during the first month of implementation. The majority of these issues involved either the firewall preventing the patient feed into PREDICT or the linking of the computer kiosk with the printer. In September 2006, focus groups were held with nurses, physicians, and information systems staff to gain additional insights into ways to improve both the tool and the process, as well as prepare for the challenges of expansion.

### Expansion phase

In February 2007, PREDICT was expanded to all patients undergoing coronary angiography within the four Kansas City metropolitan hospitals of the Saint Luke's Health System. During the expansion phase of the project, planning meetings were held at each facility prior to introducing PREDICT. Approaches to staff education, IT issues (internet access, printer connectivity, log-on assignments, etc.) were thoroughly discussed and planned for by the original implementation team with system-level staff joining the sessions. The system-level cardiovascular nursing educator (JL) was actively involved to ensure uniform implementation and a single standard of care. Leaders and staff at each of the three satellite hospitals were fully informed through staff meetings to describe the new consent form and process prior to initiation. Members of the original implementation team supported staff at each of the three suburban hospitals by on-site presence and accessibility via pager and phone. Based on feedback from the initial experience, a significant upgrade was introduced in March 2007. This included the automatic incorporation of lab data, the restructuring of data entry fields (*e.g*., anemia, chronic renal insufficiency) and the introduction of a Spanish version of the consent document.

Many of the issues that arose during the expansion phase were anticipated because they had already been encountered during the pilot phase (*e.g*., pop-up blockers preventing the creation of the consent form, computer kiosks not communicating with printers), and solutions required collaboration with the information technology staff at each satellite hospital. However, the most significant challenges arose from expanding the process to the inpatient units. Reorganization of patient flow from the inpatient units to the holding unit was required to minimize the delay into the catheterization lab (*i.e*., patients were 'called for' earlier). In addition, cardiovascular fellows had to be trained on the PREDICT process to provide consent to patients in the Cardiac Intensive Care Unit, as these patients did not flow through the holding room prior to catheterization.

### Evaluation of implementation

Overall, the implementation of the PREDICT-enhanced consent form was accomplished by employing a multidisciplinary team of clinicians and non-clinicians who understood the conceptual goal of the project and thus were able to navigate all of the anticipated and unanticipated barriers. Visible support from senior nursing and physician leadership allowed the team to work directly with the bedside staff. Information systems leaders and staff also were active in the implementation phase and now serve as the first-line troubleshooters for the ongoing maintenance of the program. In further analyzing the initial PREDICT experience, we identified two major themes: 'facilitators' and 'barriers' to implementing PREDICT consent forms. Facilitators were described as components or processes that were positive initiators and sustainers of the project. Barriers were those components or processes that were negatively received or viewed as obstacles.

### Facilitators to implementation

Facilitators to implementation included clinicians who found usefulness in the tool by providing clarity and educational value for patients. Clinician comments included 'more of my patients are reading the consent form now', 'my patient said he finally understood a form about his health', and 'my patient said she had wondered what a stent really was and now she knew'. Several physicians commented that their patients seemed better informed about the procedure and appeared less anxious. Both nursing and physician staff commented that the gathering of the patients' characteristics resulted in a thorough review of the patient's readiness for the procedure and was beneficial to clinical care.

Hospital administrators were also identified as facilitators, in that they valued the ability of PREDICT to ensure an improved standard of care related to consent forms for cardiovascular patients. They subsequently expressed the desire to convert all consent forms to a similarly easy to read, educational format.

From the perspective of the nursing staff, an important implementation facilitator was their satisfaction with the minimal data entry and minimal time required to generate the forms. The time required to collect the 18 variables ranged from four to nine minutes. The PREDICT user interface evolved based on feedback from the staff, such as auto-populating required laboratory values and determining 'presence of anemia' based on the lab value instead of requiring manual interpretation, both of which began in March 2007. This resulted in a user interface that further limited the time required by the staff to enter data, while also reducing potential errors. Drop-down menus were also introduced into the interface so that items that had been manually entered, such as physician name and procedure type, also decreased the staff's time in generating the consent. Throughout our observations of the implementation process, the staff frequently remarked that patients had a positive response to PREDICT, which enhanced and reinforced the staff's satisfaction with the new consent form and process.

The patient's experience, assessed through structured questionnaires post-procedure, demonstrated positive value with the PREDICT enhanced consent form. As compared with the original consent form, a greater percentage of PREDICT patients reported reading the consent form (PREDICT vs. original consent: 72% vs. 44%, p < 0.001), reported not feeling nervous at all after reading the consent form (65% vs. 45%, p = 0.009) and felt involved in the decisions regarding the procedure (67% vs. 45%, p = 0.003) [34].

### Barriers to implementation

The majority of identified barriers were related to information systems issues. The implementation of the new technological decision aid was accompanied by several learning opportunities, including: allowing access to secure HTTP through firewalled ports thereby permitting external access to patient data; and daily data transfers of all patients admitted. When an update was made to the main servers, we learned it was important for the central information technology staff to also update PREDICT so the website links would continue to function. Additionally, establishing secure user accounts that were adequately password protected for appropriate staff members and backup and archiving procedure development were needed. The implementation team was able to troubleshoot many issues and triaged the rest into 'internal to the hospital' issues versus 'PREDICT server' issues with appropriate solutions. A training manual was developed for new nurses and fellows and the user interface has continued to be upgraded to make the process as user-friendly as possible.

Additional barriers were reported by a subset of clinicians. A few interventional cardiologists expressed frustration at delayed patient flow into the catheterization lab and excessive patient questions, although these issues resolved rapidly over time. Several physicians commented that 'patients are asking more questions' and 'the questions are more specific,' all of which occurred before the patient was comfortable signing the form. These questions ranged from clarifying details about the logistics of the procedure to questions regarding the type of stent to be placed. While this was philosophically supported, it was operationally frustrating for these physicians. Many physicians expressed concern regarding the accuracy of the risk estimates. In one case, two physicians felt that the models assigned greater mortality risk to a patient than they perceived clinically. This required a specific physician-to-physician response to enhance their confidence in the risk percentages being predicted so the project would be unanimously accepted and adopted. Confidence in the restenosis models was even more difficult to achieve. Even though pre-procedural models are just as good in predicting restenosis as models that incorporate the results of the angiogram [20,21], the interventionalists did not readily accept this. Comments were made such as 'there is so much important data gained from the angiogram...that in many ways neutralizes the true effectiveness of the PREDICT consent form' and 'the estimates of restenosis are helpful, however, I think (at least I) incorporate many of the clinical drivers already into my decision of which stent to use.' After the process became more seamless and less intrusive, the physicians were more supportive of the project. In a survey of the interventionists in August 2007 (one year into the implementation process), all physicians responded that they would recommend the PREDICT system to colleagues around the country.

## Conclusion

This initial experience suggests that successful change in clinical processes and organizational culture can be accomplished, but requires coordination of multiple disciplines. The successful integration of a research-based, statistically driven health decision aid into routine clinical practice demonstrates the feasibility of improving the transparency of the informed consent process. Furthermore, the evolution of what had been a perfunctory, uninformative process into a well-received informed consent process that provided value to all stakeholders is a promising insight. However, the ability to change culture required the staff to embrace a different approach to patient involvement and decision-making, a paradigmatic shift for our institution. Our experience highlights the importance of local champions who could see the project through difficult times. Based upon its initial success, further expansion of PREDICT using the ePRISM^® ^technology to other cardiovascular procedures with identified risk models and to other surgical disciplines is currently being pursued. Expanding ePRISM^® ^into other key processes that require patient understanding such as discharge education is in development. Given that legal and risk management administrators have fully embraced the tool, its expansion into other areas will likely be facilitated and supported institutionally. While a potential limitation to the PREDICT and ePRISM^® ^tool evaluation is that it was performed by the developers of this new technology, developers are generally in the best position to describe the tool and the impact of the initial implementation. To ensure that future implementation and evaluation of PREDICT is minimally biased, objective, larger scale, multi-center studies with independent investigators are being planned.

Reflecting on this initial success has underscored the importance of identifying leaders and champions of change in the clinical setting. The numerous issues that arose, from 'system unavailable' to forgetting passwords, from printer problems to 'missing values needed to generate the models', required a multidisciplinary approach to overcome these inevitable obstacles. Moreover, developing personal relationships with the clinicians using the enhanced consent documents was critical to this successful pilot implementation and subsequent expansion of PREDICT to the three other system hospitals.

The PREDICT tool resulted in a transparent quantification of patients' risk profiles and an ease of communication between clinicians and patients of complex material. These preliminary data about the value of PREDICT from both clinicians' and patients' perspectives lays the foundation for a clinical trial to establish the utility of this information therapy solution to the Institute of Medicine's challenge to develop and evidence-based, patient-centered healthcare system.

## Abbreviations

PREDICT: Patient Refined Expectations for Deciding Invasive Cardiac Treatments; ePRISM^®^: electronic Personalized Risk Information Services Manager; PCI: percutaneous coronary intervention.

## Competing interests

Drs. Spertus and Soto have a patent pending on the ePRISM technology. They served as consultants to the implementation of PRISM but were not involved in the collection of data nor the analyses.

## Authors' contributions

CD participated in the design and conduct of the implementation project, data acquisition and analysis, and writing the manuscript. SVA participated in the data analysis and wrote the manuscript. OO participated in the acquisition of data and analysis. HA participated in the design and conduct of the implementation project and data acquisition and analysis. EG was champion for the product, participated in the conduct of the implementation project, data acquisition, and writing the manuscript. JL and LR were champions for the product and participated in the conduct of the implementation project. TD participated in the conduct of the implementation project. GES led development of the information technology infrastructure, designed the website, and supported the implementation. JAS provided senior leadership during all aspects of the product development, implementation, and evaluation, provided critical feedback on the manuscript, and secured funding for the project.

## Appendix

### PREDICT/ePRISM^® ^Development and Technical Specifications

1. Multidisciplinary team (cardiologists, nurses, patient interviewers, psychologists, computer informatic specialists, computer technology specialists, and statisticians) formed three subgroups (statistical, computer informatics, and human interface) to develop an information technology infrastructure for delivering predictive risk models useful to clinicians and understandable by cardiac patients.

2. Throughout the development of the program, substantial thought and insight into the requirements of a system capable of disseminating and updating a range of predictive models occurred. Relevant features include:

a) A graphical user interface for implementing prediction formulae derived from statistical risk models.

b) Dynamically generated data entry screens that allow virtually unlimited versatility with respect to potential models.

c) Models that can be accessed from a broad range of web-capable devices.

d) Model updates immediately accessible to users, allowing rapid dissemination.

Model outputs, including graphical displays, informed consent documents, and educational materials, able to be created instantly and shared with patients.

3. Multiple patient focus groups and patient interviews were conducted to ascertain what information and what output formats are most valuable to patients at the time of cardiac catheterization.

4. Preliminary risk-adjustment models of health status outcomes for PCI & coronary artery bypass graft patients were created.
